# The Needs of Orthopaedic Patients in Discharge Planning

**DOI:** 10.5704/MOJ.2211.007

**Published:** 2022-11

**Authors:** H Muhamad, MSB Yusoff, AA Shokri, Z Sulaiman, RS Bakar, NM Zain

**Affiliations:** 1 Department of Medical Education, Universiti Sains Malaysia, Kubang Kerian, Malaysia; 2 Department of Orthopaedics, Universiti Sains Malaysia, Kubang Kerian, Malaysia; 3 Women Health Development Unit, Universiti Sains Malaysia, Kubang Kerian, Malaysia; 4 Department of Psychiatry, Universiti Sains Malaysia, Kubang Kerian, Malaysia; 5 Department of Nursing, Universiti Sains Malaysia, Kubang Kerian, Malaysia

**Keywords:** discharge planning, patient needs, total knee replacement therapy

## Abstract

**Introduction:**

Patients' transition from hospital to home could be challenging for patients and caregivers. This is of utmost importance for patients requiring special or long-term care such as post-orthopaedic surgery. Effective discharge planning is required to ensure that patients are prepared to and get continuous care after returning home to prevent complications. Patients' need assessment is essential to develop effective discharge planning to meet the patient's needs.

**Materials and methods:**

This mixed-method study aimed to determine the patient's needs to develop a discharge planning for total knee replacement surgery. The needs for 96 total knee replacement patients were assessed using the Needs Evaluation Questionnaire (NEQ). The in-depth interview primary focus was to explore the lived experience of the post-total knee replacement patients receiving care in the hospital.

**Results:**

A total of 96 participants (100%) completed the NEQ questionnaire. Most of the needs concerned by the participants were expressed by at least 70% of them except the financial need (59.4%). The semi-structured interview found two elements which were a support group and patients’ needs in terms of emotional, physical and spiritual preparation in developing effective discharge planning.

**Conclusion:**

This study clarified that the patient needs assessment in the patient care plan.

## Introduction

Patients' transition from hospital to home could be a challenge for both patients and caregivers, especially in cases requiring specific or long-term care such as after invasive surgeries and those suffering are from chronic diseases. Without proper preparation, patients might develop complications due to mismanagement. As a result, the patients will be re-admitted unexpectedly due to secondary complications. Studies have shown that effective discharge planning encourages patients to go home and proceeds with good home care^1,2^. Several studies reported that an effective discharge planning system plays an important role in improving the quality of patient care, such as patients’ satisfaction^3^, caregiver’s satisfaction^4^, reduced length of hospital stay and readmission rates^5,6^ and improved functional status^7,8^.

A few studies from Malaysia also highlighted the importance of discharge planning. Research on clinical pathways reported that patients’ discharge plans improved care delivery processes and practices from the clinician through a multidisciplinary team^9^. Another study conducted among registered nurses in the Critical Intensive Care Unit (CICU) in four hospitals demonstrated the need for the discharge planning process in terms of ongoing nursing education about patients' transition experience and transitional care practice for nurses in Malaysia^10^. A survey conducted on patients and healthcare providers found that patients faced problems after being discharged to continue with home care, such as delay in the training of using crutches, prescription of medication and a few other issues. These problems might be due to the ineffectiveness of discharge planning.

Realising the advantages of the discharge planning system in the health management system, assessing the applicability and effectiveness of the discharge planning protocol are needed in the Malaysian context. This system is expected to provide high-quality care in a timely and cost-effective manner that is tailored to local needs. In building a comprehensive discharge planning system, assessing patients' needs could help the discharge planning process and provide health professionals with the specific information relating to individual patients about their requirements at this potential disjuncture between health and social care services^11^. The needs assessment of health status, expectations, and perception in patient-centred care can evaluate the care plan's current health status and process. Exploration of the need for care can help the providers and patients anticipate the level of care to be provided. Thus, the objectives of this study were to explore the needs of total knee replacement patients as a part of the element in developing discharge planning programme at a hospital.

## Materials and Methods

A cross-sectional survey was conducted on the participants through quantitative and qualitative measures among total knee replacement patients who received treatment from Hospital Universiti Sains Malaysia and Hospital Raja Perempuan Zainab (II). The quantitative measure was carried out through a questionnaire survey using the needs evaluation questionnaire (NEQ)^12^. The qualitative measure was performed through a phenomenology study using the in-depth interview. The in-depth interview primary focus was to explore the lived experience of the post-total knee replacement patients receiving care in the hospital.

Purposeful sampling was applied for both quantitative and qualitative approaches in this study. The sampling method was used due to the limited number of patients undergoing total knee replacement surgery in Kelantan. All patients who met the inclusion and exclusion criteria were invited to participate in this study.

The sample size for the quantitative part was calculated based on the Krejcie and Morgan (1970) table on sample size calculation^13^. The required sample size was estimated as 96 patients based on the total population of patients (n=120) who underwent total knee replacement in Kelantan in 2014 from both hospitals (data source from operation list book at the two hospitals). For the qualitative part, a total of 10 participants were interviewed. This study followed general rules on sample size for a qualitative interview and saturated point of data. A sufficient sample size has been reached when the same stories, themes, issues, and topics emerged from the respondents^14^.

The study protocol was approved by the Research Ethics Committee (Human) and the National Medical Research Registration [NMRR-15-1589-25150 (IIR)] and consent from the Director of Hospital. Written consent from respondents was taken to verify that they agreed to participate in the study. All participants and their backgrounds were kept anonymous and confidential.

In the quantitative part, a set of questionnaires consisting of two sections was applied to collect data regarding socio-demographic profiles and patient needs in receiving care. Section A contained socio-demographic profiles, which were gender, age, race, religion, position, marital status and other medical problems. Section B consisted of the Need Evaluation Questionnaire (NEQ), which was asked about patient needs in receiving hospital care.

The NEQ was developed by Tamburini *et al* (2000) for repeated hospitalised cancer patients. It is a useful clinical tool for obtaining a systematic and undistorted overview of the principal needs concerning patients' state of health. This instrument can also be administered by persons outside the health care system such as volunteers and incorporated in the patient hospital charts. In addition, it could be used by the medical staff to identify the actual patient needs at an early stage^12^. This instrument is frequently used to measure the relationship between patient and caregiver team from the aspect of information security needs and dialogue from the type of treatment. It is a self-reporting instrument comprising 23 items. Each item is rated on a “Yes-No” rating scale. It consists of four factors: informative firstª factor, informative secondᵇ factor, communicative factor, and relational factor. The English version of the instrument is validated using confirmatory factor analysis. The results showed that the internal consistency coefficients are presented in (Table I). A pilot study was conducted on post total knee replacement patients from Hospital Universiti Sains Malaysia (n=30) to test the questionnaire in a local setting. The Cronbach's alpha values for the scales ranged from 0.70 to 0.90, whereas the value generated from the NEQ domain was 0.900. These results supported the validity of NEQ as a research tool for this study.

**Table I:** Confirmatory factor analysis of internal consistency coefficients

Latent factors	Informative first^ª^ factor	Informative second^ᵇ^ factor	Communicative factor	Relational factor
Informative firstª factor	1.00			
Informative second^b^ factor	0.91	1.00		
Communicative factor	0.88	0.84	1.00	
Relational factor	0.49	0.45	0.50	1.00
Cronbach’s alpha	0.72	0.79	0.81	0.69

In the qualitative pat, the in-depth interview approach was utilised to ensure the same general areas of information were collected from each respondent while allowing some freedom and adaptability in obtaining the required information^15^. These procedures were adopted in this study as to explore patient needs and experience in receiving care after total knee replacement surgery.

Quantitative data was analysed using Statistical Package for the Social Sciences software (SPSS) version 22 [IBM Corp., Armonk, NY]. Descriptive analysis was used to analyse the socio-demographic and the response of patients’ needs. On the other hand, qualitative data was analysed using thematic analysis that focused on examining themes within data that required both explicit and implicit ideas^16^. In this study, the researcher used the codifying and categorising process for thematic analyses described by Saldana (2009)^17^.

## Results

A total of 96 patients completed the quantitative questionnaire, 10 of them agreed to participate in the qualitative interview session. This survey involved 96 TKR patients from various backgrounds. Table II shows that most of the patients were above 60 years old (75%) with different health conditions such as diabetes (28.1%), heart disease (6.3%), lung disease (3.1%), and other diseases (40.6%). They were from various educational backgrounds, but most had higher secondary school certificates (SPM) (76%). The majority of the patients were either pensioners or not working (77.1%), lived with a partner (53%), children (23%), and others (15.6%), whereas only 9.4% of them lived alone. Overall, most of them were living with the family members (79.2%).

**Table II:** Socio-demographic characteristics of the respondent in quantitative part (n=96).

Characteristic	N (%)
Gender	
Male	53 (55.20)
Female	43 (44.80)
Race	
Malay	96 (100)
Age	
< 60	24 (25.00)
60 – 70	61 (63.52)
> 70	11 (11.45)
Disease	
Heart disease	6 (6.30)
Diabetes	27 (28.10)
Kidney	0
Lung	4 (4.16)
Others	39 (40.60)
Education level	
SPM	45 (46.90)
Diploma	13 (13.50)
Degree	14 (14.60)
Others	24 (25.00)
Occupation	
Government	19 (19.80)
Non-government	2 (2.08)
Others	74 (77.08)
Stay with	
Partner	53 (55.20)
Alone	9 (9.40)
Children	23 (24,00)
Others	11 (11.40)

Table III shows that the socio-demographic characteristics of 10 TKR patients participated in the qualitative interview. All of them were married, nine were Malay (90%) and one was Chinese (10%). Eight of the patients were housewives (80%) and one each was a pensioner (10%) and a businessman (10%). The mean age of respondents was 56 years old and all of them were diagnosed with knee osteoarthritis.

**Table III:** Socio-demographic characteristics of the respondents in qualitative part (n=10).

No	Pseudonym	Gender	Age	Occupation
R1	RY	F	56	Housewife
R2	MA	F	63	Housewife
R3	SS	F	64	Housewife
R4	HJ	M	69	Pensioner
R5	GAK	M	69	Business
R6	HA	F	64	Housewife
R7	SSA	F	64	Housewife
R8	FA	F	60	Housewife
R9	HH	F	70	Housewife
R10	SY	F	67	Housewife

A total of 96 participants (100%) completed the NEQ questionnaire. Most of the needs concerned by the participants were expressed by at least 70% of them except the financial need (59.4%) (Table IV). The finding suggests that the importance of patient needs assessment in the patient care plan. Therefore, it should be included in the discharge planning system.

**Table IV:** The responses for patients' needs evaluation (n=96).

Variable	Response	
	Yes (%)	No (%)
I need more information about my illness	92.7	7.3
I need more information regarding my future situation (healing and the functionality of my members)	96.9	2.1
I need more information related to the examination done to me example an radiograph report	91.7	8.3
I need more information about the treatment I received example medicines	91.7	8.3
I have to be given a choice in making decisions regarding the treatment I will receive	87.5	10.4
I need specialist doctors and nurses to give me more understandable information	94.8	5.2
I need a specialist doctor to be more sincere with me	86.5	12.5
I need to have a better dialogue with my doctor	86.5	13.5
I need to receive less information about my illness (diagnosis, treatment, evolution)	22.9	77.1
I need to be less involved in decision-making	24.0	75.0
I want symptoms (pain, nausea, and so on) that I experience can be greatly reduced.	84.4	15.6
I need help in terms of eating, dressing, and going to the bathroom, especially during pain after surgery	81.3	18.8
I need a better honour than the relationship of a spouse/family member	83.3	15.6
I need a more friendly and caring nurse	90.6	8.3
I have to be more convinced by a specialist	93.8	6.3
I need better service from the hospital (bathroom, food, cleaning)	84.4	15.6
I need financial help	59.4	40.6
I need psychological support from a specialist	79.2	20.8
I need to talk to the religious adviser in performing the prayers e.g. prayers etc.	72.9	26.0
I need to talk to people with this experience	82.3	16.7
I need to be introduced to patients with this experience by a specialist doctor or nurse	77.1	21.9
I need support from my relatives	87.5	12.5
I need to feel valued in my family	90.6	8.3
I need to feel paid attention	87.5	11.5
I need to feel more independent	91.7	8.3
I need to know every needed information and requirements in advance	95.8	4.2
I need a pre-surgical clinic to be more mentally and physically fit	79.2	20.8

Two main themes emerged through the semi-structured interview, describing the patient's personal experience with total knee replacement surgery. Table V summarises the themes and sub-themes of the findings.

**Table V:** The themes and sub-themes findings from the interview

Themes	Sub-themes
1. Support Group	a. Lack of support
	b. Lack of awareness
	c. Alternative medicine
	d. Stigma
2.Patients' needs	a. Emotional preparation
	b. Physical and spiritual preparation

### 1. Support group

This theme arose from the patients' opinions regarding the support needed to perform the surgery and post-surgical care. The sub-themes addressed were lack of support from communities, lack of awareness, mythos about the surgery and alternative medicine, and stigma on other people talking about the failure of the surgery.

#### 1a. lack of support

Some of the patients in this study delayed the surgery due to the anxiety. So, the meeting had been done with other successful patient to support them, the patients decided to do the surgery. Most of the patients clearly expressed that they chose to do the surgery after meeting others who were successful in the surgery. For example, three patients stated that:

*I'm having knee pain for quite a long time but scared to do the operation. After I met a friend who recovered successfully after undergoing a total knee replacement and she always encourages me to do it… [R1, RY]*

*I’ve been having knee pain for 20 years. A doctor suggested doing it ten years ago, but I'm scared…I heard a lot of negative feedbacks… some of the patients cannot walk after the surgery, no significant improvement and others. After going to the hospital and meeting with patients who underwent successful total knee replacement and they shared their experiences that the pain was relieved after the operation and no more pain killer was needed. After that, I have decided to do the operation [R2, MA]*

*I decided to do the operation after I met with patients who underwent total knee replacement and they recovered and were able to walk. After that, I discussed with my family, and they agreed to do it. [R6, HA]*

#### 1b. Lack of awareness

Two patients suggested providing an awareness campaign to all the communities. They believed that if the community knew about the outcome of the surgery from the healthcare providers and the experience of the successful patients, they would willingly present/undergo for the surgery.

*Before I did the operation, there was no class@course like this. It is good to have a course@class like this so that we are aware of it. [R2, MA]*

*If possible, health care people should go to the mosque because I have friends suffering from knee pain, but they are scared to go to the hospital. [R6, HA]*

#### 1c. Alternative treatment

Seeking alternative medicine was common among the community. Most of the patients were taking other alternative medicine before deciding to do the surgery as mentioned by these patients;

*Every time I go to get a shaman service but still do not get well, rather it becomes worse… [R1, RY]*

*I've spent a lot of money to see the shaman to cure my knee pain. However, I’m confused because there is a famous shaman that just asked me to drink spell water for my knee treatment ... it does not heal ...I only spend money ... when the knee becomes bad ... [R5, GAK]*

*I also went to see a shaman and ate the alternative medicine, but it did not heal … [R9, HH]*

#### 1d. Stigma

Failure in operation information and experiences from other patients gave a stigma in performing the surgery. Three of the participants raised the issue;

*I have been having knee pain for almost two and a half years…scared to do the operation, heard that people could not walk after the operation… [R2, MA]*

*For the first time, I'm scared to do the operation… all friends who came to visit said that they could not perform daily activities after the operation… [R3, SS]*

### 2. Patients' needs

This theme arose from patients' opinions about their needs for preparation before and after the surgery. The sub-themes addressed were emotional, physical and spiritual preparation.

#### 2a. Emotional preparation

Anxiety is a common problem for patients undergoing surgery. Below were some of the statements from the participants indicating the need for emotional preparation.

*Fear of being unusual for operation and misery for children to guard ... I am sick and it is too difficult because the frequent calling for the nurse would later trouble them… [R2, MA]*

*For sure I'm afraid because I have no idea of what is going to happen…, All children are working and going back when the surgery is completed... [R3, SS]*

#### 2b. Physical and spiritual preparation

Most of the participants were Malay and they were worried about postural changes in performing prayer after the surgery. They expected that they could perform prayer as usual in a normal posture after the surgery.

*Can I pray as usual after the operation? [R4, HJ]*

*What happens if this operation just makes me feel more pain, and I still cannot perform prayer… [R7, SSA]*

*How about my function in performing prayers, will it become normal or not? [R8, FA]*

## Discussion

The patient’s needs assessment was emphasised in this study because it was an important element to build a comprehensive discharge planning system. An assessment of patients’ needs could help the discharge planning process and provide health professionals with the specific information relating to individual patients about their requirements and needs in receiving care services^18^, evaluate current health status and process of the care plan^19^, and help the providers and patients anticipate the level of care to be provided^20^.

Patients in this study clearly expressed their needs and concerns in receiving care. Majority of the patients agreed that they need more information about their illness, future situation, examination and treatment. They also need an effective therapeutic relationship between doctors and nurses, such as effective communication. Supporting and caring from relatives, health care professionals, patient groups, and other related persons were highly agreed by the patients.

Two themes were identified through the interviews with the patients. Particularly, the themes expressed by the patients were the support and also their needs. These two themes are so closely interrelated, and it may be plausible to assume that if their individual needs for reassurance and advice were met, this could address some of their worries about their sickness.

Patients felt lack of support in terms of people who have the same problem that could encourage them to receive the hospital treatment, lack of awareness about the benefit of receiving proper treatment and influenced by the mythos which better seeking for alternative medicine than receiving hospital treatment. Patients also have the stigma that most cases become worse for post-operation than before the surgery.

A support group is a group of people or individuals with a common experience and concern who provide emotional and moral support for one another (Meriam Webster dictionary). Offering patients access to support groups may help address their psychosocial needs^21^, facilitate open conversation on patients' fear, concern, preference, needs, improve knowledge and quality of life^22^. A peer may provide a dimension of care different from a physician who has never experienced the disease^21^. In this study, the patient support group became the patient's need to improve the patient knowledge, quality of life and facilitate open conversation on patients' anxiety and confidence level. Patients would gain the information through the experience exchanged and share between them^21^, sharing the illness experience might lead to the improved coping mechanism and decrease the prevalence of anxiety^23^. From these study findings, the strategies that should be included in the development of discharge planning are specific information, referrals to local support groups and services, and adequately addressing anxieties specific to improving patient discharge planning^2^.

This study has some weaknesses that limit our interpretation and create opportunities for future research. Firstly, this study was confined to only two hospitals in Kelantan, limiting the generalisability of the findings. However, the results explained the needs of patients who underwent total knee replacement in receiving care. Secondly, a small sample size of 96 patients underwent a total knee replacement. Therefore, the result might not mirror the actual consequence of the survey on patient needs but based on the total population of patients (n=120) that underwent total knee replacement in 2014. The sample size for this study was estimated as 96 patients based on the Krejcie and Morgan table on sample size calculation for a finite population.

## Conclusion

This study on needs assessment has highlighted the areas where total knee replacement patients from Kelantan believe that they have needs and unmet needs. It has also shown that the critical needs of the patient in receiving care. The development of discharge planning should consider the individual needs of patients to achieve optimal patient care, prevent complications and improve patient’s care quality.
